# 2610. Home Administration of Intranasal Live Attenuated Influenza Vaccine: A Review of Current Evidence and Potential for Meeting Vaccination Targets

**DOI:** 10.1093/ofid/ofad500.2224

**Published:** 2023-11-27

**Authors:** Ravi Jhaveri, Matt Zahn, Allyn R Bandell

**Affiliations:** Ann & Robert H. Lurie Children's Hospital of Chicago, Chicago, Illinois; Orange County Health Care Agency, Santa Ana, California; BioPharmaceuticals Medical, AstraZeneca, Gaithersburg, Maryland

## Abstract

**Background:**

During the 2010–2021 influenza seasons, vaccination coverage was below the 70% target for the United States at 33.4–58.6% among children and 50.2% among adults.[1, 2] The live attenuated influenza vaccine (LAIV) is currently approved for healthcare professional administration (HCPA); however, self- and home-administered LAIVs may provide an additional, effective option for vaccine administration, which could expand seasonal influenza vaccine accessibility. LAIV is a quadrivalent influenza vaccine administered intranasally, with comparable effectiveness to the inactivated influenza vaccine.[3] Broadening vaccine availability to multiple vaccination sites has been shown to increase vaccination rates by up to 22%.[4]

Here we summarize the current evidence on self- and home-administered LAIV efficacy and safety, and potential applications for increasing community influenza vaccination accessibility.

**Methods:**

Publications evaluating the efficacy, safety, and practicality of self- and home-administered LAIV were identified from a literature search with the following search string: (live attenuated influenza vaccine OR LAIV) AND (home administration OR self administration OR self immuni*).

**Results:**

In three studies comparing LAIV self- and home administration to HCPA (**Table**), home administration was preferred by 64, 74, and 96% of participants. Sample sizes ranged from 41 children to 4561 patients receiving LAIV, with participants aged between 2.6 and 64 years old. A trial of 4561 patients comparing LAIV self- and home administration to HCPA groups found similar effectiveness against influenza-like illness, immunogenicity, and adverse events post-vaccination.

**Figure:**
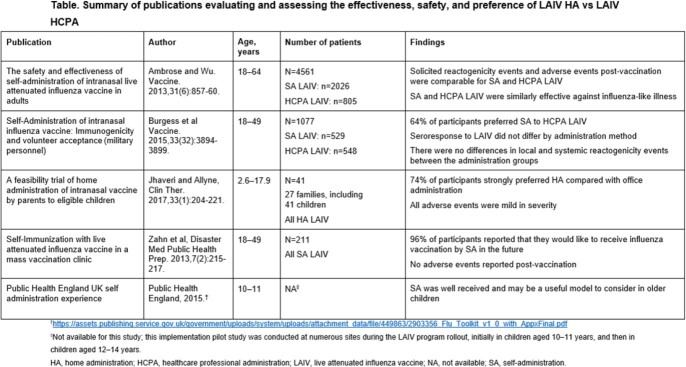

**Conclusion:**

Evidence suggests that self- and home-administered LAIV could be safely and effectively offered, and may be preferable for a subset of the population, which could expand seasonal influenza vaccine accessibility.

**References**

[1] Centers for Disease Control and Prevention. Flu vaccination coverage, United States, 2020–21 influenza season.

[2] Gates DM et al. Vaccine. 2022;40(44):6337-6343.

[3] Bandell A et al. Open Forum Infect Dis. 2020;7(Suppl 1):S709.

[4] Ipsos. Expanding flu vaccine access.

**Disclosures:**

**Ravi Jhaveri, MD**, AbbVie: Grant/Research Support|AliOS: Grant/Research Support|AstraZeneca: Advisor/Consultant|Gilead: Grant/Research Support|MedImmune: Advisor/Consultant|Merck: Grant/Research Support|Saol Therapeutics: Advisor/Consultant|Seqirus: Advisor/Consultant **Allyn R. Bandell, PharmD**, AstraZeneca: employee|AstraZeneca: Stocks/Bonds

